# Ambient air pollution and psoriasis: a nationwide cross-sectional study of 149 744 Chinese patients in 31 provinces

**DOI:** 10.7189/jogh.16.04010

**Published:** 2026-02-27

**Authors:** Lingbo Bi, Ziyi Wang, Jungang Yang, Ziyuan Tian, Hanqing Zhao, Zining Xu, Kejun Chen, Zhou Zhuang, Xiaoyi Huang, Hongfei Ouyang, Yujun Sheng, Yong Cui

**Affiliations:** 1 Department of Dermatology, China-Japan Friendship Hospital, Beijing, China; 2 Graduate School, Beijing University of Chinese Medicine, Beijing, China

## Abstract

**Background:**

Skin is the largest organ of the human body. It continuously encounters environmental toxicants, including airborne pollutants, which may induce many skin disorders, such as psoriasis. However, evidence on the association between airborne pollutants and psoriasis prevalence in China remains limited.

**Methods:**

We used nationwide inpatient diagnostic data on psoriasis from 2021 to 2023, encompassing 149 744 cases across 31 provinces, municipalities, and autonomous regions in China, along with corresponding air pollution data. We analysed the spatial distribution and clustering patterns of psoriasis using the spatial autocorrelation analysis. We employed Pearson correlation analysis and Geodetector to explore the spatial heterogeneity of psoriasis and its association with airborne pollutants at the provincial level. We assessed the explanatory power of individual airborne pollutants and their combined effects on psoriasis prevalence.

**Results:**

Pearson correlation analysis revealed that PM10 (r = 0.604), PM2.5 (r = 0.429), air quality index (AQI) (r = 0.542), and NO_2_ (r = 0.476) have significant positive correlations with psoriasis prevalence. Psoriasis and its subtypes exhibited significant spatial heterogeneity and diverse clustering patterns across regions. Geodetector identified PM10 (q = 0.357; *P* = 0.000), AQI (q = 0.315; *P* = 0.000), and O_3_ (q = 0.264; *P* = 0.000) as key contributors to this spatial heterogeneity. Interactive detection analysis further revealed that the combined effects of specific pollutant pairs, including PM2.5 and SO_2_ (q = 0.790), PM10 and SO_2_ (q = 0.727), as well as O_3_ and SO_2_ (q = 0.704), played a pivotal role in explaining the prevalence of psoriasis. The other combinations also showed an important impact on psoriasis subtypes, including psoriasis vulgaris (PM2.5 and SO_2_) (q = 0.792), psoriasis erythematous (PM2.5 and SO_2_) (q = 0.852), psoriatic arthritis (PM10 and O_3_) (q = 0.840), and nail psoriasis (PM10 and O_3_) (q = 0.789).

**Conclusions:**

The airborne pollutants influence psoriasis prevalence and its subtypes. With the largest global study of the Asian population, we provide novel insights into the impact of air pollution on psoriasis, guiding future public health policies and clinical interventions.

Psoriasis is a chronic, immune-mediated systemic disease with cutaneous manifestations. It represents a significant global health burden due to its high prevalence, chronicity, and profound impact on quality of life [1,2]. Worldwide, psoriasis affects over 60 million people, with regional variations influenced by genetic, environmental, and lifestyle factors [3]. In China, as one of the most common autoimmune diseases, the prevalence of psoriasis is estimated at 0.47% which means at least six million individuals live with the disease [4,5]. Epidemiological studies have highlighted significant spatial heterogeneity in psoriasis prevalence, suggesting that environmental factors, including climate and air quality, may contribute to disease distribution and severity [6,7].

The relationship between skin disorders and environmental factors, such as climate, has been reported [8,9]. Previous studies revealed that cold, dry climates may be associated with increased disease severity [10,11]. Humidity and UV levels have been reported to regulate skin dryness and psoriasis-related inflammation [12]. However, the role of airborne pollutants, functioning as a critical environmental factor in rapidly industrialising regions such as China, remains poorly understood. Airborne pollutants, including fine particulate matter (PM2.5), inhalable PM (PM10), nitrogen dioxide (NO_2_), sulphur dioxide (SO_2_), and ozone (O_3_), have been implicated in the pathogenesis of various inflammatory and autoimmune diseases, such as asthma, cardiovascular disease, and rheumatoid arthritis [13–16]. There is also evidence supporting that exposure to airborne pollutants may increase the risk of oxidative stress and inflammation in psoriasis [17,18]. Yet, their specific impact on psoriasis incidence and severity has not been systematically investigated, particularly in China’s diverse geographic and environmental landscape.

This study represents the first comprehensive analysis of the association between psoriasis incidence, including its subtypes, and airborne pollutant levels across all provinces, autonomous regions, and municipalities in China. By integrating detailed epidemiological data from 2021–2023 with high-resolution air quality measurements, we aimed to investigate the environmental determinants of psoriasis. The novelty of our study lies in the granular, region-specific analysis and in the focus on interactions among multiple air pollutants and psoriasis subtypes. Furthermore, we leveraged advanced spatial statistical methods to elucidate the complex interplay between environmental factors and disease distribution.

## METHODS

### Data resource and processing

We included disease, population, and air quality data. We obtained psoriasis diagnosis data from 2021–2023, including subtypes, from the China National Centre for Quality Control of Skin and STDs, which dermatologists across China determined according to the Chinese guidelines for psoriasis diagnosis and treatment. The psoriasis prevalence was calculated using the number of cases and population data from the China Statistical Yearbook. We calculated provincial air pollution averages from city-level data published by the Atmospheric Composition Analysis Group at Dalhousie University.

To reduce potential bias from other social factors, we stratified the 31 provinces, autonomous regions, and municipalities into three layers based on the China Development Index published by Renmin University of China in 2022 [19]. Based on the data from the China Statistical Yearbook 2022, Renmin University of China contains indexes from six aspects, including health, life quality, education, social environment, economy, and developing capacity, which comprehensively reflect the development at the provincial levels (Table S1 in the **Online Supplementary Document**).

### Pearson correlation analysis and stratification

We performed the Pearson correlation analysis in *R*, version 4.4.3 (R Core Team, Vienna, Austria). We stratified 31 provinces, autonomous regions, and municipalities into three layers based on their ranks at Renmin University of China. Layer one had the highest rank, indicating that the provinces, autonomous regions, and municipalities were the most developed regions in China during this period.

### Spatial autocorrelation analysis

We performed spatial autocorrelation analysis, including global and local autocorrelation, using the built-in Spatial Statistics module in ArcGIS, version 10.8 (Esri, Redlands, California).

Global autocorrelation describes the overall distribution of the study object at the spatial level and indicates whether it exhibits spatial clustering. We chose the most used index for investigation:



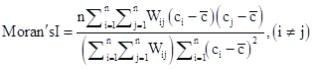



In the formula, n represents the number of study units, c_i_ and c_j_ denote the psoriasis incidence rates of the i-th and j-th study units, respectively. c̄ is the average psoriasis incidence rate across all study units, and Wij is the spatial weight matrix.



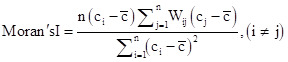



Local spatial autocorrelation is used to examine the spatial relationship between a region's psoriasis incidence rate and its neighbouring areas. The formula is as follows:

In the formula, n also represents the number of study units, c_i_ and c_j_ denote the observed value of psoriasis incidence rates of the i-th and j-th study units, respectively. c̄ is the average psoriasis incidence rate across all study units, and Wij is the spatial weight matrix.

### Instruction spatial weight matrix

We constructed a spatial weight matrix to reveal spatial associations among geographical objects, requiring prior definition of spatial adjacency relationships before conducting a spatial autocorrelation analysis. The creation of an n × n normalised spatial adjacency matrix effectively represents the spatial location of individual objects or the proximity relationships between regions. The fundamental form of the normalised spatial adjacency matrix can be expressed as:



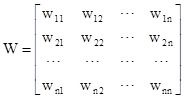



In the formula, Wij represents the adjacency degree between region i and j.

### Standard deviational ellipse

The standard deviational ellipse is a classical algorithm that simultaneously analyses the direction and distribution of points and reflects the spatial distribution characteristics of geographic elements. The major axis of the ellipse indicates the direction of data distribution, while the minor axis represents the extent of data distribution. The greater the difference between the lengths of the major and minor axes, the more pronounced the directionality of the data distribution. The formula is as follows:



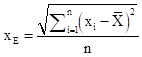





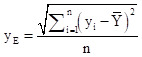





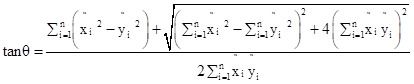





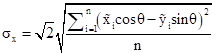





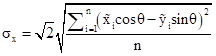



In the formula: x_e_ and y_e_ are the coordinates of the centre of the standard deviational ellipse, x_i_ and y_i_ are the centroid coordinates of each study unit, X̄ and Ȳ are the average centroid of all study units, n is the number of study units, θ is the azimuth angle of the standard deviational ellipse, x̃_i_ and ỹ_i_ represent the coordinate deviations of each study unit's centroid from the average centroid, and σ_x_ and σ_y_ are the standard deviations of the x-axis and y-axis, respectively.

Based on the ellipse model analysis, the major semi-axis characterises the dominant trend direction of the data, while the minor semi-axis reflects the spatial diffusion range. The ellipticity (ratio of major to minor semi-axis) indicates the strength of directional features: a higher ellipticity (ratio >1) corresponds to more pronounced directional characteristics, whereas when the ratio approaches one, the data exhibits isotropic distribution. A shorter minor semi-axis suggests enhanced centripetal clustering, whereas its elongation indicates increased dispersion.

### Geodetector

The geodetector is a statistical method that explores the spatial heterogeneity of research objects based on data stratification and reveals the underlying driving factors [20]. It includes factor detection and interaction detection. The calculation formula is as follows:



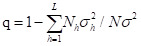



In the formula, q represents the explanatory power of the influencing factor on the psoriasis incidence rate. The larger the q-value, the stronger the explanatory power of the indicator on the spatial distribution of psoriasis incidence (q∈[0,1]). L is the number of categories of the influencing factor, N_h_ and σ_h_^2^ denote the sample size and variance of the h-th category of the influencing factor, respectively, while N and σ^2^ represent the total number of study units and the variance of the psoriasis incidence rate, respectively.

### Selection of influencing factor parameters

The air quality index (AQI) is a quantitative measure of air quality. According to China's new air quality evaluation standards released in March 2012, the pollutant monitoring indicators include SO_2_, NO_2_, PM10, PM2.5, carbon monoxide (CO), and O_3_. Based on the graded concentration limits for each pollutant, the individual air quality sub-indices are calculated from measured concentrations of PM2.5, PM10, SO_2_, NO_2_, O_3_, and CO (where PM2.5 and PM10 are 24-hour average concentrations). The AQI is determined by selecting the maximum value from the individual AQIs of these pollutants.

## RESULTS

### Descriptive results

There were 149 744 inpatient cases diagnosed with psoriasis from 1 January 2021 to 31 December 2023, according to the China National Centre for Quality Control of Skin and STDs. We also extracted statistics of 15 533 patients who were diagnosed with psoriatic arthritis (PsA), 166 with psoriasis erythematous (PE), 405 with nail psoriasis (PsN), and 131 297 with psoriasis vulgaris (PsV).

### Pearson correlation analysis

**Figure 1:**
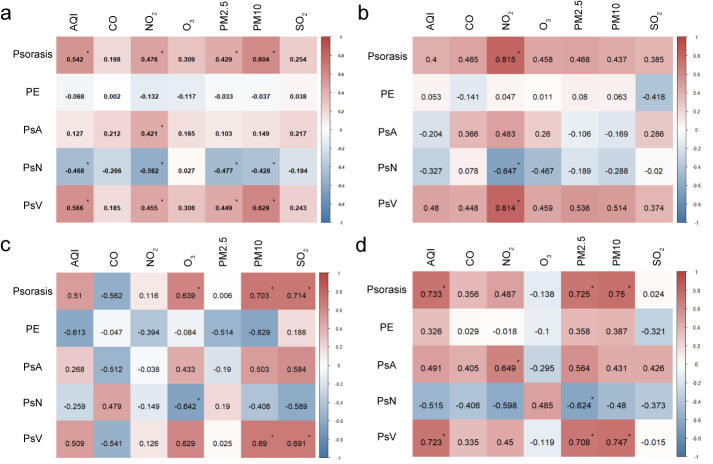
Pearson correlation analyses on the prevalence of airborne pollutants and psoriasis and its subtypes. **Panel A. **Psoriasis. **Panel B.** PsV** Panel C.** PsN** Panel D.** PsV. *P < 0.05. AQI – air quality index, CO – carbon monoxide, NO_2_ – nitrogen dioxide, O_3_ – ozone, PM2.5 – fine particulate matter, PM10 – inhalable particulate matter, SO_2_ – sulphur dioxide, PE – psoriasis erythematous, PsA – psoriatic arthritis, PsN – nail psoriasis, PsV – psoriasis vulgaris.

Psoriasis was positively correlated with all environmental factors, and most correlations were statistically significant except CO and SO_2_ (**Figure 1**, Panel A). Among these, PM10 had the highest correlation coefficient (r = 0.604), followed by AQI (r = 0.542).

Among the four psoriasis subtypes, PsN showed a negative correlation with all factors except for O_3_. The strongest negative correlation was also with NO_2_ (r = −0.562). PsV was positively correlated with all environmental factors, with PM10 as the most significant contributor (r = 0.629).

### Pearson correlation analysis in subgroups

We stratified the 31 provinces into three layers, with development increasing from layer one to layer three. In layer three, which included most of the developed regions in China, including Beijing, Shanghai, Jiangsu, Guangdong, and Zhejiang, NO_2_ showed the most significant correlation with psoriasis (r = 0.815) and PsV (r = 0.814) (**Figure 1**, Panel B)

In layer two, however, the correlation with psoriasis was led by SO_2_ (r = 0.714) and PM10 (r = 0.703). However, PsN was negatively correlated with O_3_ (r = −0.642) (**Figure 1**, Panel C). In layer one provinces and autonomous regions, PM10, PM2.5, and AQI showed significant positive correlations with psoriasis and PsV (**Figure 1**, Panel D).

### The spatial distribution characteristic of psoriasis incidence

#### Coexistence of regional aggregation and punctate concurrence

**Figure 2:**
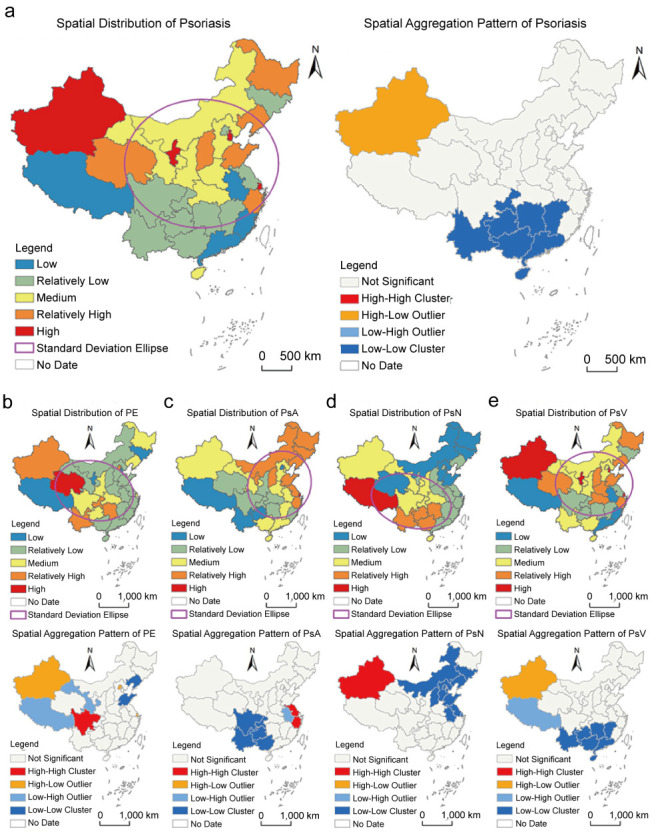
Spatial distribution and agglomeration patterns of psoriasis and its subtypes. **Panel A.** Psoriasis. **Panel B.** PE. **Panel C.** PsA. **Panel D.** PsN. **Panel E.** PsV. PE – psoriasis erythematous, PsA – psoriatic arthritis, PsN – nail psoriasis, PsV – psoriasis vulgaris.

Based on the natural breaks method, we divided the psoriasis incidence rate into five levels (*i.e.* low, relatively low, medium, relatively high, and high). There were four provinces/municipalities at the high level, namely Xinjiang, Ningxia, Tianjin, and Shanghai, which were sporadically distributed among the eastern, northern, and northwestern regions (**Figure 2**, Panel A). Five provinces/municipalities had a relatively high level, showing a relatively dispersed spatial distribution. Provinces with a medium level were relatively clustered, concentrated in the central and northern regions, and connected the high and relatively high areas. The relatively low areas were more concentrated, mainly distributed in the regions south of the Yangtze River. Provinces with low levels were distributed across relatively low areas. From the standard deviational ellipse, the centre of psoriasis incidence was in North China, with an overall slight east-west distribution. In general, the psoriasis incidence rate exhibited a distribution characteristic of regional agglomeration with higher rates in the north and lower rates in the south, alongside sporadic coexistence.

#### Prominent spatial disparity between the north and the south

The results of global spatial autocorrelation showed that the global Moran’s I for psoriasis incidence is 0.046, which is significant at the 95% confidence level, indicating the presence of spatial clustering in the distribution of psoriasis incidence. Further local autocorrelation analysis revealed three patterns in the spatial distribution of psoriasis incidence in China: high-low clustering, low-high clustering, and low-low clustering (**Figure 2**, Panel A). Among these, high-low clustering and low-high clustering were only seen near Xinjiang and Tibet, respectively, while low-low clustering was distributed in a clustered pattern at the junction of South China and Southwest China. This demonstrates a significant spatial differentiation pattern of higher psoriasis incidence in the north and lower incidence in the south in China.

#### Spatial distribution characteristics of incidence rates for psoriasis subtypes

We also classified the incidence rates of various psoriasis subtypes into five levels (low, relatively low, medium, relatively high, and high) using the natural breaks method.

#### PE type shows a northwest-southeast distribution along the second terrain step

The high-value area of PE type included only Qinghai Province (**Figure 2**, Panel B). The relatively high-value areas consisted of four regions: Xinjiang, Yunnan, Hunan, Chongqing, and Beijing. The medium-value areas were located between the high-value and relatively high-value areas, connecting them to form a clustered region. The relatively low-value areas were concentrated in South China, the eastern coastal regions, and North China, while the low-value areas included only Tibet, Jilin, Ningxia, and Tianjin, scattered sporadically. The distribution centre was located in the southwestern region, showing a significant shift toward the southwest compared to the overall distribution. The distribution was notably northwest-southeast, indicating a substantial change from the overall pattern. The global autocorrelation results showed that the global Moran’s I for PE-type psoriasis incidence was 0.007, indicating weak spatial clustering. Further local spatial autocorrelation analysis showed four types of clustering for PE-type psoriasis incidence: high-high, high-low, low-high, and low-low. Overall, this shows a spatial pattern of higher rates in the west and lower rates in the east.

#### PsA type exhibits a northeast-southwest spatial gradient differentiation trend

The high-value area of PsA type included only Shanghai (**Figure 2**, Panel C). The relatively high-value areas were mainly distributed in Northeast China, and in Ningxia, Shanxi, Shandong, Jiangsu, and Zhejiang. The medium-value areas formed an inverted C-shape, isolating the low and relatively low-value areas from the relatively high-value areas. The relatively low-value areas were primarily distributed in the central and western regions, while the low-value areas were in the southwestern region, presenting an overall gradient differentiation pattern with higher rates in the northeast and lower rates in the southwest. From the standard deviational ellipse, the PsA type generally followed a southeast-southwest distribution. The global Moran’s I for PsA type was 0.093, which was significant at the 95% confidence level, indicating significant spatial clustering. Further local autocorrelation results (**Figure 2**, Panel C) revealed that the spatial clustering of PsA type includes high-high, low-low, and high-low clusters. Among these, the high-high cluster was located in the Yangtze River Delta region, while the low-low cluster was in the southwestern region, showing an overall differentiation trend between the northeast and southwest.

#### PsN type shows significant gradient differentiation along the altitude-based first, second, and third terrain levels

Due to China's terrain distribution, it is often divided into three parts from high to low: the higher-altitude (First Terrain Step), the lower-altitude (Third Terrain Step), and the middle-altitude (Second Terrain Step). PsN type had only one high-value area, located in Tibet on the first terrain step (**Figure 2**, Panel D). On the second terrain step, only Qinghai and Inner Mongolia were relatively low-value areas, while the rest were medium- or high-value areas. The relatively high-value areas were clustered in the southwestern region, showing significant gradient differences across the first, second, and third terrain steps. Overall, the distribution followed a northwest-southeast direction, with the distribution centre located in the southwestern region. The global Moran’s I was 0.185, which was significant at the 99% confidence level, indicating notable spatial clustering. Local autocorrelation results showed that the spatial clustering of PsN type consisted only of high-high patterns. Among these, the high-high cluster was limited to Xinjiang, while the low-low cluster was centred around Beijing, forming a block-like aggregation in North China and the eastern coastal regions. The disparity between higher rates in the west and lower rates in the east was pronounced.

#### Spatial distribution of PsV type highly overlaps with the overall pattern of psoriasis incidence

The high-value and low-value areas of PsV type mostly aligned with the overall distribution of psoriasis incidence (**Figure 2**, Panel E). Differences were observed in only five provinces/regions: Hubei and Henan were classified as relatively high-value areas, while Yunnan, Guangxi, and Beijing were medium-value areas. From the standard deviational ellipse, the centre of psoriasis incidence was in North China, with an overall slight east-west distribution. In general, the psoriasis incidence rate exhibited a distribution characteristic of regional agglomeration with higher rates in the north and lower rates in the south, alongside sporadic coexistence. The global spatial autocorrelation results showed that the global Moran’s I for PsV type was 0.048, which is significant at the 95% confidence level, indicating spatial clustering in the distribution of PsV type. There were three clustering patterns: high-low, low-high, and low-low. Among these, the high-low cluster included only Xinjiang, the low-high cluster included only Tibet, and the low-low cluster forms a block-like distribution at the junction of South China and Southwest China. The spatial differentiation pattern of higher rates in the northwest and lower rates in the southeast was prominent.

#### Factor detector evaluated the influence of various driving factors on the spatial differentiation of psoriasis and its subtypes

We used the factor detector to obtain the q-values representing the influence of various driving factors on the spatial differentiation of psoriasis and its subtypes and ranked them accordingly (**Table 1**).

**Table 1:** Factor detection results for psoriasis and subtypes

	Total			PE			PsA			PsN			PsV		
	**q**	***P*-value**	**Rank**	**q**	***P*-value**	**Rank**	**q**	***P*-value**	**Rank**	**q**	***P*-value**	**Rank**	**q**	***P*-value**	**Rank**
**AQI**	0.315	0.000	2	0.106	0.000	2	0.056	0.022	7	0.280	0.000	2	0.342	0.000	2
**CO**	0.109	0.000	7	0.067	0.008	4	0.068	0.008	5	0.046	0.043	7	0.102	0.000	7
**NO_2_**	0.215	0.000	4	0.044	0.051	5	0.144	0.000	2	0.256	0.000	3	0.212	0.000	5
**O_3_**	0.264	0.000	3	0.004	0.882	7	0.136	0.000	3	0.067	0.009	6	0.256	0.000	3
**PM2.5**	0.211	0.000	5	0.085	0.000	3	0.223	0.000	1	0.177	0.000	4	0.219	0.000	4
**PM10**	0.357	0.000	1	0.156	0.000	1	0.101	0.000	4	0.284	0.000	1	0.374	0.000	1
**SO_2_**	0.132	0.000	6	0.013	0.487	6	0.057	0.018	6	0.105	0.000	5	0.129	0.000	6

AQI – air quality index, CO – carbon monoxide, NO_2_ – nitrogen dioxide, O_3_ – ozone, PM2.5 – fine particulate matter, PM10 – inhalable particulate matter, SO_2_ – sulphur dioxide, PE – psoriasis erythematous, PsA – psoriatic arthritis, PsN – nail psoriasis, PsV – psoriasis vulgaris

For psoriasis, all influencing factors passed the significance test, and their explanatory power was ranked as: PM10>AQI>O_3_>NO_2_>PM2.5>SO_2_>CO, showing that PM10, AQI, and O_3_ were the primary factors influencing the spatial distribution differences of psoriasis. Combined with the positive correlation coefficients found in Pearson correlation analysis, this suggests that poorer environmental quality is associated with higher psoriasis incidence.

We further investigated the influence of various driving factors on the spatial differentiation of psoriasis subtypes.

For the PE subtypes, the ranking of factors with significant explanatory power was PM10>AQI>PM2.5>CO>NO_2_, showing that PM10, AQI, and PM2.5 were the main influencing factors for PE type. The negative correlation coefficients, however, suggested that poorer environmental quality may be associated with lower PE-type incidence, indicating a complex mechanism of environmental factors on PE type.

Similarly, all influencing factors showed a significant explanatory power on PsN, ranking as PM10>AQI>NO_2_>PM2.5>SO_2_>O_3_>CO. Combined with the negative correlation coefficients, this suggests that poorer environmental quality is associated with lower PsN-type incidence.

However, for PsA, all influencing factors showed significant explanatory power, ranking as PM2.5>NO_2_>O_3_>PM10>CO>SO_2_>AQI, with positive correlation coefficients, indicating that poorer environmental quality is associated with higher PsA incidence.

For the PsV, all influencing factors passed the significance test, and their explanatory power was ranked as PM10>AQI>O_3_>PM2.5>NO_2_>SO_2_>CO, suggesting that poorer environmental quality is associated with higher PsV-type incidence.

#### Analysis of interactive detection results

**Figure 3:**
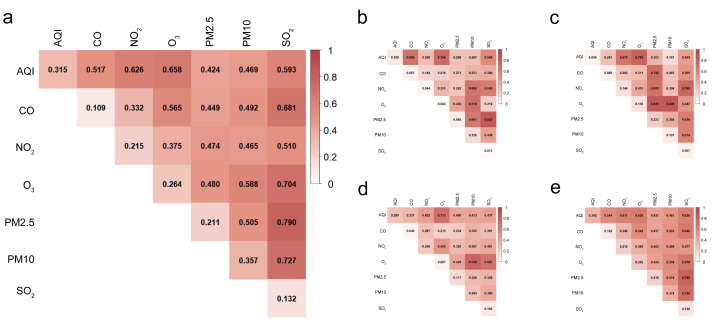
Spatial distribution and aggregation patterns of incidence rates of four different psoriasis subtypes. **Panel A.** Psoriasis. **Pabel B.** PE. **Panel C.** PsA. **Panel D.** PsN. **Panel E.** PsV. AQI – air quality index, CO – carbon monoxide, NO_2_ – nitrogen dioxide, O_3_ – ozone, PM2.5 – fine particulate matter, PM10 – inhalable particulate matter, SO_2_ – sulphur dioxide, PE – psoriasis erythematous, PsA – psoriatic arthritis, PsN – nail psoriasis, PsV – psoriasis vulgaris.

Among the factors influencing psoriasis, the q-values for the interaction of SO_2_ with PM2.5, PM10, O_3_, and CO were relatively large and significantly higher than the individual q-values of each interacting factor (**Figure 3**, Panel A). This indicates that the spatial distribution of psoriasis is primarily influenced by the interaction of multiple factors, particularly the SO_2_.

We further investigated the effect of interaction detection across psoriasis subtypes. For the PE type, the interaction q-values for SO_2_ and PM2.5, AQI and O_3_, PM10 and O_3_, CO and AQI, as well as PM10 and NO_2_ were relatively large and significantly higher than the individual q-values of each single factor (**Figure 3**, Panel B).

When it came to the PsA, the interaction q-values for PM10 and O_3_, PM2.5 and O_3_, AQI and O_3_, SO_2_ and NO_2_, as well as PM2.5 and NO_2_, were relatively large and showed stronger q-values than those of each single factor (**Figure 3**, Panel C).

For PsN, the interaction q-values for PM10 and O_3_, O_3_ and AQI, SO_2_ and O_3_, O_3_ and NO_2_, as well as NO_2_ and AQI were significantly higher than the individual q-values of each single factor (**Figure 3**, Panel D).

Among the interaction effects of factors for the PsV type, the interaction q-values for SO_2_ and PM2.5, SO_2_ and PM10, SO_2_ and O_3_, SO_2_ and CO, as well as O_3_ and AQI were relatively large and significantly higher than the individual q-values of each single factor (**Figure 3**, Panel E).

## DISCUSSION

Skin is the largest organ in the human body. It is exposed to many extrinsic factors, including UV radiation, smoking, humidity, as well as airborne pollution, all the time [21]. Although the airborne pollution effects on respiratory and cardiac health and diseases have been repeatedly discussed, the potential effect of airborne pollution on skin disorders has not been comprehensively addressed in China [22]. Therefore, we conducted a comprehensive analysis of the relationship between psoriasis incidence, including its subtypes, and air pollutant levels across China from 2021–2023. By integrating epidemiological data with high-resolution air quality measurements, we identified significant associations between specific air pollutants and psoriasis prevalence, as well as notable differences in how various psoriasis subtypes respond to environmental factors.

In our study, psoriasis and its subtypes showed both shared and distinct responses to different types of air pollution. For overall psoriasis incidence, PM10 (q = 0.356) and AQI (q = 0.311) emerged as the most influential factors, suggesting that poorer air quality is associated with higher disease prevalence. Two recent studies also supported the psoriasis-promoting effect of PM10 [23,24]. However, by analysing long-term follow-up data from the UK Biobank, these two studies also revealed that exposure to other air pollutants, such as PM2.5 and NO2, significantly increases the risk of psoriasis, a finding not validated in our study. It is speculated that this discrepancy may be due to differences in racial composition between our study population (mainly Asian) and the UK Biobank (94.22% were European Caucasians).

Different from PM10, AQI is a crucial and commonly used indicator that describes the overall quality of air, which integrates several pollutants, including PM2.5, PM10, O_3_, NO_2_, SO_2_, and CO [25]. A population-based cohort study reported an apparent dose-dependent association between AQI and rosacea, another inflammatory skin disorder [26]. The air with different AQI can cause various levels of skin thickening in mice after dissolving in water, which is a classic pathological manifestation of psoriasis [27]. However, we are the first to reveal a direct, robust association between AQI and psoriasis prevalence, suggesting that multiple pollutants may collectively contribute to the increased incidence of psoriasis, with PM10 playing a particularly significant role.

Although only PM10 and AQI showed a strong connection with the heterogeneous spatial distribution of psoriasis, further investigation revealed more associations between other air pollution indicators and psoriasis subtypes. As the most common subtype of psoriasis, PsV type mirrored the overall psoriasis pattern. PM10 (q = 0.374) and AQI (q = 0.342) had the highest explanatory powers, suggesting heightened sensitivity to air quality in this subtype [28]. PM2.5 (q = 0.223) and NO_2_ (q = 0.144), however, exhibited strong explanatory power in PsA type, suggesting that fine PM and NO_2_ play a significant role in driving this subtype. A time-series analysis has reported that NO_2_ showed significant and stable effects on the outpatient visits for psoriasis [29]. As a subtype of psoriasis, PsA leads to more severe pathological manifestations that extend beyond the skin to involve the joints, occurring in approximately 30% of patients with psoriasis [30]. With the systemic inflammatory effects, NO_2_ has also been reported to exacerbate autoimmune arthritis [31]. The same effect of NO_2_ may also be seen in systemic inflammation in PsA.

In the following analysis of interactive detection results, the interaction between factors showed significantly greater explanatory power for psoriasis and its subtypes than did each factor. The improved explanatory power was obvious, especially for O3 and PM2.5, as well as for AQI and SO2, the interaction of which showed a significantly higher explanatory power for differences in psoriasis, PsV, and PE incidence, respectively, although the explanatory power of each factor alone was weak. In other studies, it has also been observed that the synergistic effects of air pollution factors may exhibit a stronger association with disease than individual factors alone [32]. The interactive detection also reported strong explanatory power for psoriasis subtypes, with the interaction between SO2 or NO2 and other airborne pollutants such as CO and O_3_. In addition to their intrinsic physicochemical properties, pollutants such as NO_2_ and SO_2_ exhibit high chemical reactivity. Previous studies suggested that NO_2_-induced lipid peroxidation disrupts skin barrier function, while SO_2_-derived sulphites may alter the pH of the stratum corneum, thereby altering the local cutaneous microbiota [33,34]. In brief, when they come into contact with human skin from the air, they may undergo chemical reactions with surface moisture and lipids, thereby altering the pH and microbiota of the exposed skin areas, which could elevate the risk of psoriasis [35]. Given that various air pollutants often co-occur, results from interactive detection more closely reflect the real-world air pollution exposure experienced by individuals at risk of psoriasis.

Using nationwide diagnostic and air quality data, we conducted a nationwide cross-sectional study that revealed a connection between airborne pollutants and psoriasis prevalence. The impact of the environment on individual health has emerged as a research area. Other studies are focusing on this topic and might employ long-term follow-up data from the UK Biobank study or cross-sectional data, but on a smaller population scale [36]. To our knowledge, this is the first epidemiological study that comprehensively evaluated the effect of exposure to environmental pollutants on psoriasis across such vast real-world populations and diverse geographical settings. The large population base in China contains psoriasis patients, accounting for nearly 10% of the global total, which provides our study with a sufficient and geographically diverse sample of psoriasis cases (149 744 inpatient cases from 2021–2023). Besides, China’s vast territory and diverse geographical features create a vibrant natural and climatic environment, while the uneven levels of industrial development and environmental governance measures result in significant variations in airborne pollutant levels across its provinces [37–40]. These factors collectively provide our study with unique advantages and enhance the credibility [41].

For the methodological design, our study employed Geodetector based on its unique advantages in the geographical detector model. This technique effectively dissects the independent contributions of multi-scale environmental exposures and identifies nonlinear synergistic effects through geographical spatial heterogeneity detection and interaction analysis, making it particularly suitable for risk factor screening in exploratory research. Compared to spatial regression models that rely on assumptions of data distribution and linear relationships, or Bayesian hierarchical models that are sensitive to prior distributions, Geodetector quantifies explanatory power (q-value) and interaction types without requiring predefined model structures, which aligns better with our research objective of revealing spatial association patterns between air pollution and psoriasis.

However, our study has some limitations. First, the data we used were based on the number of inpatients with psoriasis and did not include outpatient cases, which may reduce the data's representativeness. We still need studies that incorporate multicentre cohort designs to complement and validate our findings through causal inference in the future. Additionally, we did not perform time-series or seasonal analyses, which could have enhanced the robustness of this study. In addition, we did not employ the data at more granular levels; a supplementary analysis could provide more information on this topic.

We established a comprehensive nationwide cross-sectional investigation integrating environmental exposure and dermatological epidemiological data. The underlying link between airborne pollutants and psoriasis or other cutaneous diseases worldwide could be investigated in the future.

## CONCLUSIONS

We identified airborne pollutants with significant connections to psoriasis incidence in China, with distinct patterns observed across subtypes. We investigated the spatial pattern of airborne pollutants and psoriasis prevalence in a country characterised by geographic heterogeneity, demographic complexity, climatic diversity, and uneven industrialisation. Our findings underscore the pivotal role of mitigating airborne pollutants in curbing psoriasis incidence, advocating the integration of environmental exposure screening into clinical management protocols for psoriasis patients.

## Additional material


Online Supplementary Document
