# Supplementary material for: Ambient air pollution and psoriasis: a nationwide cross-sectional study of 149 744 Chinese patients in 31 provinces
Source: J Glob Health. 2026 Feb 27;16:04010. doi: 10.7189/jogh.16.04010 (PMC12967242; doi:10.7189/jogh.16.04010)
Supplement: Online Supplementary Document [file jogh-16-04010-s001.pdf]

Supplement to: Bi L, Wang Z, Yang J, Tian Z, Zhao H, Xu Z, Chen K, Zhuang Z, Huang X, Ouyang H, Sheng Y, Cui Y. Ambient air pollution and psoriasis: a nationwide cross-sectional study of 149 744 Chinese patients in 31 provinces. J Glob Health. 2026;16:04010.

Supplementary Table 1. Rank of provincial regions based on the RCDI(Renmin University of China released the China Development Index) in 2022.

Region name	RCDI	RCDI stratification
Beijing	100.28	3
Shanghai	93.36	3
Jiangsu	91.1	3
Guangdong	90.93	3
Zhejiang	89.91	3
Shandong	86.11	3
Tianjin	85.14	3
Fujian	84.85	3
Hubei	83.9	3
Anhui	83.45	3
Chongqing	83.07	2
Hunan	82.68	2
Inner Mongolia	82.68	2
Jiangxi	82.53	2
Shaanxi	82.26	2
Qinghai	80.77	2
Henan	80.75	2
Hebei	80.65	2
Sichuan	80.58	2
Ningxia	80.3	2
Jilin	79.95	1
Xinjiang	79.83	1
Tibet	79.73	1
Liaoning	79.4	1
Heilongjiang	79.28	1
Shanxi	79.26	1
Guizhou	79.14	1
Guangxi	79.09	1
Hainan	78.37	1
Yunnan	78.37	1
Gansu	78.33	1
